# Fabrication of curcumin-loaded magnetic PEGylated-PLGA nanocarriers tagged with GRGDS peptide for improving anticancer activity

**DOI:** 10.1016/j.mex.2023.102229

**Published:** 2023-05-23

**Authors:** Fatih Senturk, Soner Cakmak

**Affiliations:** a Department of Biophysics, Faculty of Medicine, Duzce University, Duzce, Turkey; b Division of Bioengineering, Graduate School of Science and Engineering, Hacettepe University, Ankara, Turkey

**Keywords:** PLGA, GRGDS, Magnetic polymeric nanoparticles, Cellular targeting, Cur loaded and GRGDS conjugated magnetic PLGA nanocarriers for enhanced anticancer activity

## Abstract

Carrier-mediated drug delivery systems are highly promising as a treatment option for the targeted delivery of potent cytotoxic drugs with increased efficacy and safety. Considering that poly (lactic-co-glycolic acid) (PLGA) and polyethylene glycol (PEG) polymers each provide certain advantages for biological purposes, PEGylated-PLGA nanoparticles have emerged as a leading candidate among other alternatives. Furthermore, these nanoparticles can be modified with the specific short peptide sequences such as glycine-arginine-glycine-aspartic acid‑serine (GRGDS), which selectively binds to integrins overexpressed in most cancer cells, allowing for targeted delivery. Here, we reported the details in fabrication and characterization of magnetic PEGylated-PLGA nanoparticles functionalized with GRGDS peptide. In addition, superparamagnetic iron oxide nanoparticles (SPIONs) and the natural pharmaceutical compound curcumin (Cur) were loaded into these polymeric nanoparticles to assess their anticancer activity potential. Overall, this study provides comprehensive methodologies, including all synthesis procedures, challenges, and useful suggestions for peptide-conjugated polymeric nanoparticles that may be used for cellular targeting and therapeutic applications.

• Step by step fabrication protocol for the Cur loaded magnetic PEGylated-PLGA nanoparticles was presented.
• Validation of the fabrication and the GRGDS conjugation to the nanoparticles were shown via detailed characterization studies.
• The cytotoxic effect of the Cur-loaded and GRGDS-tagged magnetic nanoparticles was tested on T98G glioblastoma cell line as a preliminary *in vitro* study.

## Graphical abstract

**Figure:**
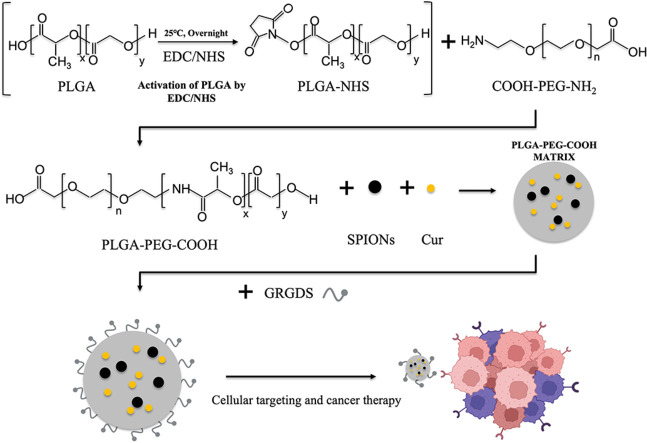

Specifications table

**Table:** 

Subject area:	Pharmacology, Toxicology and Pharmaceutical Science

More specific subject area:	Targeted drug delivery
Name of your method:	Cur loaded and GRGDS conjugated magnetic PLGA nanocarriers for enhanced anticancer activity
Name and reference of original method:	SPIONs, J. Park, K. An, Y. Hwang, J.-G. Park, H.-J. Noh, J.-Y. Kim, J.-H. Park, N.-M. Hwang, T. Hyeon, Ultra-large-scale syntheses of monodisperse nanocrystals, Nature Materials 3(12) (2004) 891–895. https://doi.org/10.1038/nmat1251 PEGylated PLGA, S. Prabhu, J.S. Goda, S. Mutalik, B.S. Mohanty, P. Chaudhari, S. Rai, N. Udupa, B.S.S. Rao, A polymeric temozolomide nanocomposite against orthotopic glioblastoma xenograft: tumor-specific homing directed by nestin, Nanoscale 9(30) (2017) 10,919–10,932. https://doi.org/10.1039/C7NR00305F Curcumin and SPIONs loaded GRGDS-peptide conjugated PEGylated PLGA nanocarriers, F. Senturk, S. Cakmak, I.C. Kocum, M. Gumusderelioglu, G.G. Ozturk, GRGDS-conjugated and curcumin-loaded magnetic polymeric nanoparticles for the hyperthermia treatment of glioblastoma cells, Colloids and Surfaces A: Physicochemical and Engineering Aspects (2021) 126648. https://doi.org/10.1016/j.colsurfa.2021.126648.
Resource availability:	All reagents indicated in this method are commercially accessible. A description of the details is provided in paper [1].

## Background

It is considered that nanoparticles are excellent carriers since they have a strong potential to carry agents directly to the tumor location, thus minimizing the risk of systemic toxicity as well as adverse effects. Nanocarriers have grown in popularity as a way of increasing therapeutic efficacy while reducing the frequency of drug administration [2]; hence their most common application is to carry therapeutics to malignancies for more effective chemotherapy. Chemotherapy is essential for the treatment of malignant tumors; however, its efficiency is typically restricted due to the poor physiochemical characteristics of anti-tumor drugs, such as low stability, short circulation half-life, and toxicity to normal tissues [3]. To overcome these limitations, it is necessary to effectively deliver therapeutics to tumor tissues while limiting their presence at other sites [4]. Because of all this, functional nanoparticles are recognized as superior carrier candidates for chemotherapeutics. In addition, nanoparticles can be also a suitable carrier matrix for natural bioactive compounds like curcumin, whose therapeutic usage is limited by their poor bioavailability and low solubility in an aqueous environment.

For many years, superparamagnetic iron oxide nanoparticles (SPIONs) have been investigated for a wide range of biological applications to take advantage of their superior magnetic characteristics. For instance, SPIONs have been used for magnetic-guided targeted drug delivery, magneto-thermal stimulation, contrast agents in magnetic resonance imaging (MRI) [5], increasing blood-brain barrier permeability in the presence of electromagnetic fields [6], and magnetic nano hyperthermia in cancer therapy [7], [8], [9]. Considering all of these applications, it is essential to identify the appropriate nanocarriers to which SPIONs and therapeutics can be transported for the success of therapy or any other missions.

Several polymers, such as poly (lactic-co-glycolic acid) (PLGA), poly (lactic acid) (PLA), polyethylene glycol (PEG), and polyvinyl alcohol (PVA), have been investigated as nanocarriers to improve the poor solubility or retention time of various agents. However, PLGA has become the most preferred polymer in the fabrication of nanocarriers because of a number of advantages, such as effective intracellular endocytosis, FDA approval, and the ability to control hydrophilicity and degradation rate using an adjustable lactide:glycolide ratio [10]. Additionally, PEGylated-PLGA provides additional functionality, such as reducing the opsonization of blood proteins, preventing macrophage recognition, and evading the immune system [1,4]. Although using PEGylated-PLGA polymers as nanocarriers has several advantages in biological applications, they should be specifically designed to target a specific cell, tissue, or organ and release their drug depot onsite.

PEGylated-PLGA nanocarriers can accumulate in tumors either by passive or active targeting. Passive targeting implies that nanoparticles can enter the tumor's leaky vascular tissue and accumulate in solid tumors through the well-known enhanced permeability retention (EPR) effect [11]. However, the pathophysiological condition of the tumor tissue is the only factor that decides whether the passive strategy can successfully transport agents to the desired area. On the other hand, active targeting can attain the desired site by molecular recognition with an appropriate ligand that can identify its receptor [3]. Thus, selective/active targeting to certain receptors of the tumor cells can be established by conjugating targeting ligands to the surface of nanocarriers such as antibodies, peptides, or aptamers [12]. Among them, peptide-based bio-probes are favored owing to their high binding affinity to cell surface receptors, low immunogenicity, and ease of chemical production [13].

The cell adhesion sequence of fibronectin, arginine-glycine-aspartic acid (RGD) tripeptide, has a high affinity for integrin proteins [14]. As integrin proteins are overexpressed in the vasculature of tumor microenvironments, RGD peptides can be immobilized on the surface of nanocarriers for targeting purposes [15]. In addition, it has been demonstrated that conjugation of the glycine-arginine-glycine-aspartic acid‑serine (GRGDS) peptide to the nanocarrier surface enhances cellular uptake [16]. Since this peptide has a selective affinity for the integrin proteins that are overexpressed in most cancer cells, we focused on GRGDS as a potential targeting peptide. There are several methods for immobilizing peptides on nanoparticle surfaces [17]. However, covalent immobilization, such as EDC/NHS chemistry, is favored in targeting systems due to their ability to maintain the activity of peptides on the nanoparticle surface for an extended period [18]. In this study, SPIONs and curcumin loaded PEGylated-PLGA nanoparticles functionalized with GRGDS peptide were fabricated and characterized in detail. Furthermore, in order to verify the efficacy of GRGDS-conjugated nanocarriers, a preliminary *in vitro* cell viability test utilizing human glioblastoma cell line (T98G) was performed. Therefore, detailed descriptions of the methods, including all synthesis processes, challenges, and practical recommendations, are provided in the following sections.

## Method details

### Preparation of SPIONs

**Fig. 1:**
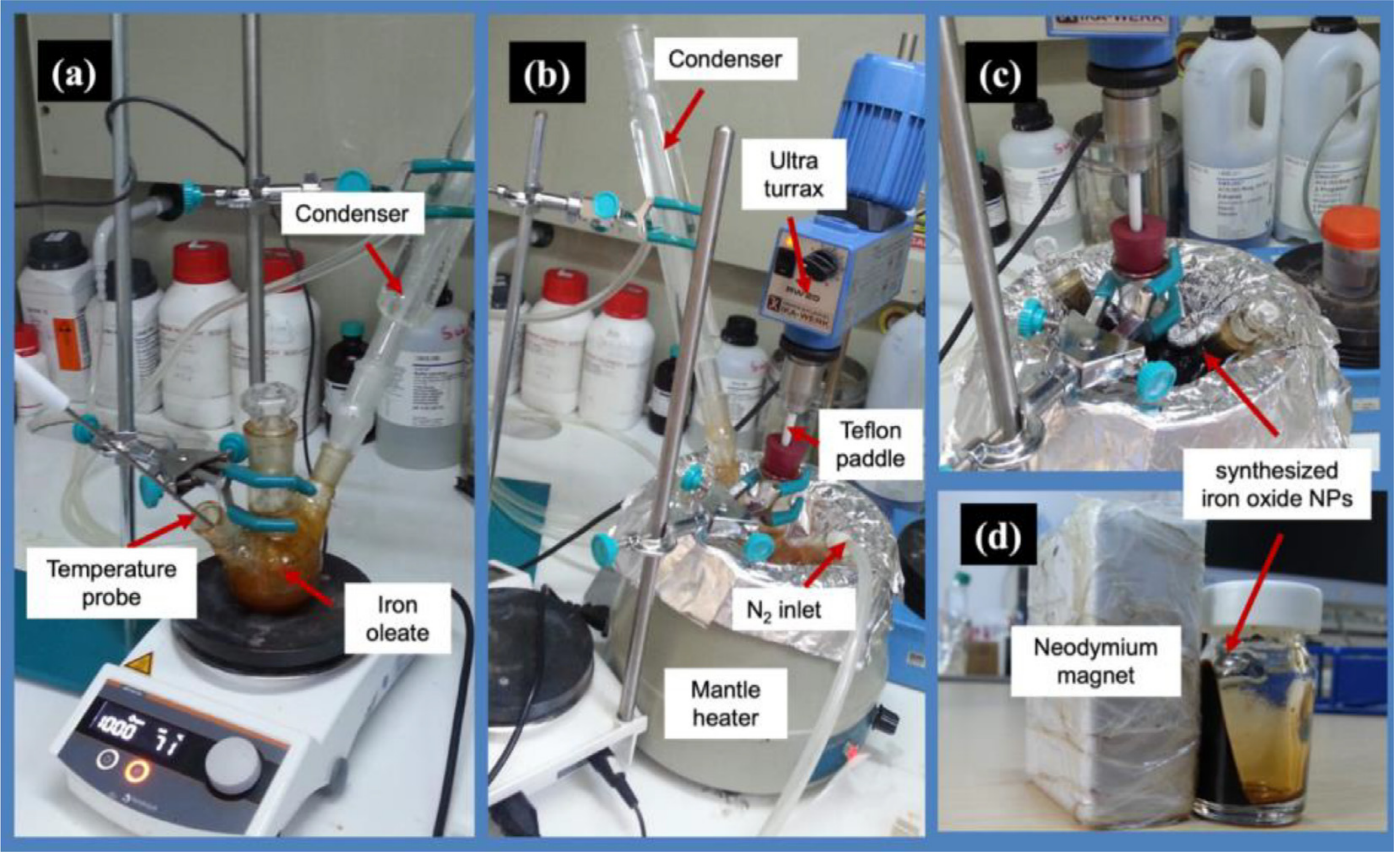
Synthesis steps of iron oxide nanoparticles via thermal decomposition method, (a) formation of the iron-oleate complex, (b) decomposition of the iron-oleate compound via controlled temperature increase, (c) formation of the black iron oxide precipitate, (d) magnetic separation of iron oxide nanoparticles with a neodymium magnet.

The thermal decomposition method was slightly modified for the fabrication of superparamagnetic iron oxide nanoparticles (SPIONs) [19]. There are two stages in the thermal decomposition method for producing SPIONs. The first step in the process involves the reaction of sodium-oleate with iron (III) chloride (FeCl_3_•6H_2_O), which results in the production of iron-oleate (Fig. 1a). In the following step, iron-oleate is thermally decomposed in a high-boiling-point organic solvent (1-octadecene) to produce SPIONs (Fig. 1b).

• The iron-oleate compound was created by dissolving FeCl_3_•6H_2_O (5.4 g) and sodium oleate (18.25 g) in 40 mL of ethanol (EtOH), 30 mL of distilled water, and 70 mL of hexane.
• The resulting mixture was heated to 70 °C and stirred for 4 h at that temperature.
• The reaction solution was then cooled to room temperature, and the upper organic layer containing the iron oleate was washed 3 times with deionized water using a separatory funnel.
• In the second step, the synthesized iron oleate (18 g) and oleic acid (2.8 g) were dissolved in 1-octadecene (100 g) under the protection of N_2_ (Fig. 1b).
• The temperature of the solution was raised to 320 °C (3 °C/min rate) and maintained for 30 min.
• After cooling the solution down to room temperature, the excess surfactants were then removed by washing the magnetic NPs with EtOH using a neodymium magnet and centrifugation (10,000 rpm, 15 min).
• The obtained magnetic NPs were distributed in hexane and kept at 4 °C.

### Magnetization hints for synthesized-SPIONs

• If the magnetic characteristics of the synthesized SPIONs are crucial for the planned study, the synthesis procedure should be handled with additional care.
• It is possible for thermal decomposition-produced SPIONs to have low saturation magnetization. For instance, magnetic nanoparticles (MNs) have a physical and magnetic diameter, and if their magnetic diameter decreases or a "magnetic dead layer" forms on their surface, the magnetization of MNs can be lowered [20,21].
• Additionally, the effective magnetic moment can be reduced by magnetic dipole-dipole interactions, which was reported as the cause of poor magnetization in several studies involving the synthesis of MNPs [20,22,23].
• There is also the fact that the magnetization value of SPIONs varies dramatically depending on whether or not O_2_ is present in the synthesis atmosphere [21].
• If the study focuses on magnetic nano hyperthermia, SPIONs with low magnetization values may also display heating efficiency, since magnetization is not the only factor determining this efficiency; low aggregation, homogenous, and uniform size distribution are also crucial [24].

### Fabrication of PEGylated-PLGA di-block copolymer

Di-block polymers of PLGA (lactide:glycolide, 50:50, Mw:30,000–60,000) and poly(ethylene glycol) 2-aminoethyl ether acetic acid (COOH-PEG-NH_2_, Mn:3500) were conjugated via an EDC/NHS (EDC: 1-ethyl-3-(3-dimethyl-aminopropyl) carbodiimide, NHS: N-hydroxysuccinimide sodium salt) chemistry [25,26].

• A mixture of 50 mg PLGA, 2.3 mg EDC, and 1.35 mg NHS was dissolved in 10 mL dimethylformamide (DMF) and stirred (100 rpm) overnight.
• The PLGA-NHS polymer was precipitated using ice-cold methanol, centrifuged at 6,000 rpm for 10 min (three times), and dried under a vacuum.
• The following step involved dissolving 40 mg of activated PLGA-NHS in 2 mL of DMF, reacting with 0.18 mg of PEG, and incubating at room temperature for 12 h.
• The resulting PEGylated-PLGA copolymer was precipitated, washed three times with ice-cold methanol, collected, and vacuum dried.
• Fabrication of the PEGylated-PLGA copolymers was validated by characterizing the chemical structure with ^1^H nuclear magnetic resonance (^1^H NMR) and Fourier Transform Infrared Spectroscopy (FTIR).

### Amine coupling hints

• Some organic bases such as triethylamine (TEA) or *N,N*-diisopropylethylamine (DIPEA or DIEA) can be used as deprotonation agents for NH_2_-PEG-COOH.
• These bases can be added to the reaction medium to enhance the conjugation efficiency between PLGA-NHS and NH_2_-PEG-COOH [27,28].
• In order to achieve optimum conjugation efficiency, a PLGA-NHS/ NH_2_-PEG-COOH molar ratio of 1:1 to 1:1.5 is recommended [27], [28], [29].

### Fabrication of curcumin-loaded magnetic PEGylated-PLGA NPs

The single emulsion-solvent evaporation method was modified to fabricate curcumin-loaded magnetic PEGylated-PLGA nanoparticles (PEG-PLGA-Cur-MNPs).

• Four mL of dichloromethane (DCM) was used to dissolve 20 mg of PEGylated-PLGA and 8 mg of curcumin by vortexing.
• The polymeric drug solution was combined with 2 mg of SPIONs and sonicated in the bath sonicator to obtain a clear organic-phase solution.
• Then, the mixture was dropped into 20 mL of 1% (w/v) polyvinyl alcohol (PVA) solution and then homogenized for 10 min at 15,000 rpm in a T25 ultra-turrax.
• Afterward, the organic solvent (DCM) was evaporated while the emulsion was mechanically stirred with a non-magnetic PTFE stirrer for 4 h at room temperature.
• Subsequently, PEG-PLGA-Cur-MNPs were collected by centrifugation (11,000 rpm) at 4 °C for 30 min and washed three times with deionized water to remove any excess reactants.
• The resulting PEG-PLGA-Cur-MNPs were frozen at −80 °C before being lyophilized and stored at 4 °C for future usage.

### Encapsulation efficieny of curcumin from NPs (direct method)

• Encapsulation efficiency (EE%) of curcumin was measured using UV spectrophotometer (Nanodrop 2000c, Thermo Scientific, USA) for both PEG-PLGA-Cur-MNPs and GRGDS-PEG-PLGA-Cur-MNPs.
• Lyophilized NPs were dissolved in acetonitrile (1 mg/mL) using an ultrasonic water bath and a magnetic stirrer.
• Neodymium magnets were used for magnetic separation to extract SPIONs, while centrifugation was used to remove impurities.
• The concentration of Cur was determined by measuring the absorbance at 420 nm and calculated via standard curve of Cur dissolved in acetonitrile (0–100 µg/mL) (Fig. 8).

### Production hints for the PEG-PLGA-Cur-MNPs

• Impurities originating from the surface breakdown of the metal ultrasonic probe infiltrate into the reaction medium during extended ultrasonication. Therefore, we propose utilizing an ultraturrax or ultrasonic bath as a homogenizer, optimizing the application time.
• We advise using a non-magnetic PTFE stir bar rather than magnetic fish or iron stirrers because the nanoparticle synthesis solution contains magnetic nanoparticles.
• Because centrifuges are used to wash the nanoparticles, and an ultrasonic bath or vortex is utilized to re-homogenize the nanoparticles after centrifugation in each washing step, the amount of encapsulated agent may be reduced in these cases.
• The encapsulation efficiency of nanoparticles can be calculated using indirect methods (from supernatant) and direct methods (from NPs). However, it should be noted that the EE% of NPs measured from the supernatant may be misleading due to the possible instability of agents in the supernatants throughout the washing processes of NPs. Therefore, the direct method (from NPs) should be utilized to calculate the EE% of NPs.

### Immobilization of GRGDS on the surface of cur-loaded magnetic PEGylated-PLGA NPs

**Fig. 2:**
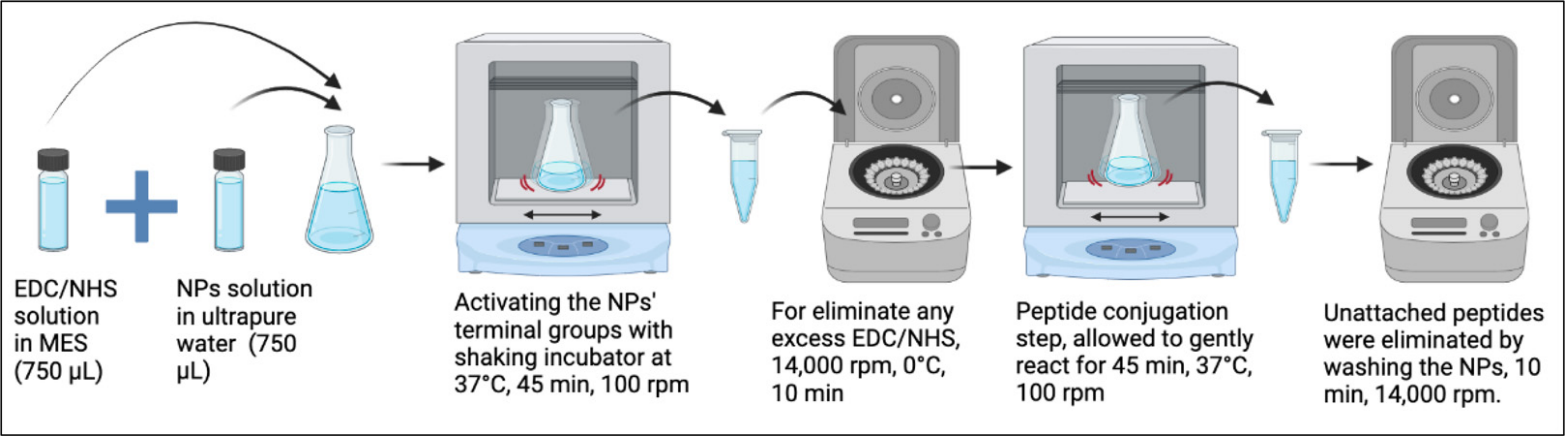
Schematic representation of the GRGDS peptide conjugation to the PLGA nanoparticles (created by bioRender.com).

The peptide molecules were synthesized using a standard solid-phase peptide synthesis method with fluorenylmethyloxycarbonyl (Fmoc) chemistry by Genecust (Luxembourg). Also, the chemical structures of the peptide molecules were engineered to include the fibronectin adhesion sequence arginine-glycine-aspartic acid‑serine (RGDS). Furthermore, a glycine amino acid was added to the RGDS peptide structure to liberate the molecule, creating the NH_2_-GRGDS-COOH amino acid sequence. Peptide-NPs conjugation was accomplished via EDC/NHS chemistry (Fig. 2).

• The EDC/NHS solution (750 µL) was prepared using 75 mg of EDC and 75 mg of NHS in 0.1 M 2-(N-morpholino) ethanesulfonic acid (MES) buffer at pH:5.
• Fifteen mg of NPs was dispersed in 750 µL of deionized water.
• The homogeneously dispersed NP solution was combined with 750 µL of EDC/NHS solution and incubated at 37 °C in a shaking incubator (100 rpm) for 45 min.
• After that, the NPs solution was centrifuged (14,000 rpm) for 10 min and washed with MES buffer (0.1 M) to eliminate any residual EDC/NHS.
• The EDC/NHS activated NPs (1 mg/mL) were redispersed in 750 µL of peptide solutions (1 mg/mL GRGDS in MES buffer at pH:5), and the peptides were then allowed to chemically conjugate to NPs (45 min, 37 °C, 100 rpm).
• After this process, unattached peptides were also eliminated by washing the NPs twice in MES buffer (10 min, 14,000 rpm).

The amount of peptide bonded to the surface of the NPs was assessed using the TAMRA-GRGDS peptide sequence, which contains the fluorescent chemical group carboxytetramethyl rhodamine (TAMRA). The TAMRA-GRGDS peptides were also conjugated to NPs using the same procedure described above. However, after the conjugation reaction of TAMRA-GRGDS was complete, the washing solutions were gathered by centrifugation, and the quantity of binding peptide was evaluated indirectly by a fluorescence spectrophotometer.

• The calibration curve was established using TAMRA-GRGDS solutions at the excitation (544 nm) and emission (572 nm) wavelengths.
• The tested TAMRA-GRGDS concentrations ranged from 0 to 20 µg/mL.
• The amount of peptide binding was calculated using the calibration curve.
• Fluorescence microscopy was also utilized to observe the TAMRA-GRGDS linked NPs.

### Hints for conjugation peptides to polymeric NPs

When the peptide conjugation procedure is applied to a drug-loaded nanocarrier, the encapsulation efficiency (EE%) of the nanocarrier typically decreases at the end of the process. These reductions are probably the result of drug leakage from the nanocarrier during the additional washing and shaking steps required for the peptide conjugation process.

### Preliminary *in vitro* cell viability test

The resulting nanoparticles were named, curcumin-loaded magnetic PEGylated-PLGA nanoparticles (PEG-PLGA-Cur-MNPs) and GRGDS-conjugated curcumin-loaded magnetic PEGylated-PLGA nanoparticles (GRGDS-PEG-PLGA-Cur-MNPs). Before experiments, human glioblastoma cells (T98G) were cultured in Dulbecco's Modified Eagle Medium (DMEM F-12) supplemented with 10% (v/v) fetal bovine serum (FBS) and 1% (v/v) penicillin-streptomycin.

• T98G cells were seeded in a 96-well plate at a density of 10^4^ cells/well and incubated at 37 °C in a humidified 5% CO_2_ environment overnight.
• Nanoparticles were exposed to UV light for 10 min to ensure their sterility and avoid contamination.
• The initial growth medium was then replaced with a new one that included different concentrations of well-dispersed NPs.
• As a control group, cells were also exposed to the same volume of medium free of NPs.
• After 72 h treatment of NPs at 0, 0.25, 0.50, and 1.0 mg/mL concentrations, the cytotoxicity of NPs on T98G cells was determined using the MTT assay.

### MTT assay

• After 72 h incubation, the medium was replaced with a fresh one containing MTT reagent to each well (20 μL/well of MTT solution at 5 mg/mL in PBS) and incubated for 3 h at 37 °C.
• After 3 h, the medium was removed, and the formazan crystals were dissolved by adding isopropanol (200 μL) into each well.
• Finally, cell viability was assessed with a microplate reader by measuring the absorbance at a wavelength of 570 nm (reference wavelength: 650 nm).

### Hints for *in vitro cell* viability test

• A homogenous dispersion of nanoparticles in the culture medium prior to treatment is crucial for further analysis.
• Long-term UV exposure may cause or accelerate PLGA degradation [30].
• The polymeric nanoparticles can also be sterilized using a 0.22 µm polyether sulfone (PES) syringe filter. However, the concentration of NPs should be diluted and optimized for sterile filtration, as the high viscosity of NPs can be problematic.
• Recently, more convenient alternatives to the MTT test have been established [31]. The water-soluble tetrazolium salt (WST) assay may be preferred as an easier option than the MTT assay as it shortens the analysis steps.

### Statistical analysis

All data were evaluated as the mean ± standard deviation (SD) with five independent replicates (*n* = 5). Statistical significance was determined via student's *t*-test. The concentration of NPs inducing a 50% inhibition of cell growth (IC_50_) was calculated graphically by nonlinear regression using the curve-fitting of GraphPad Prism 9.5.0 (GraphPad Inc., USA). *P* value less than 0.05 indicated statistical significance (*p<0.05, **p<0.01, ***p<0.001).

### Methods validation

**Fig. 3:**
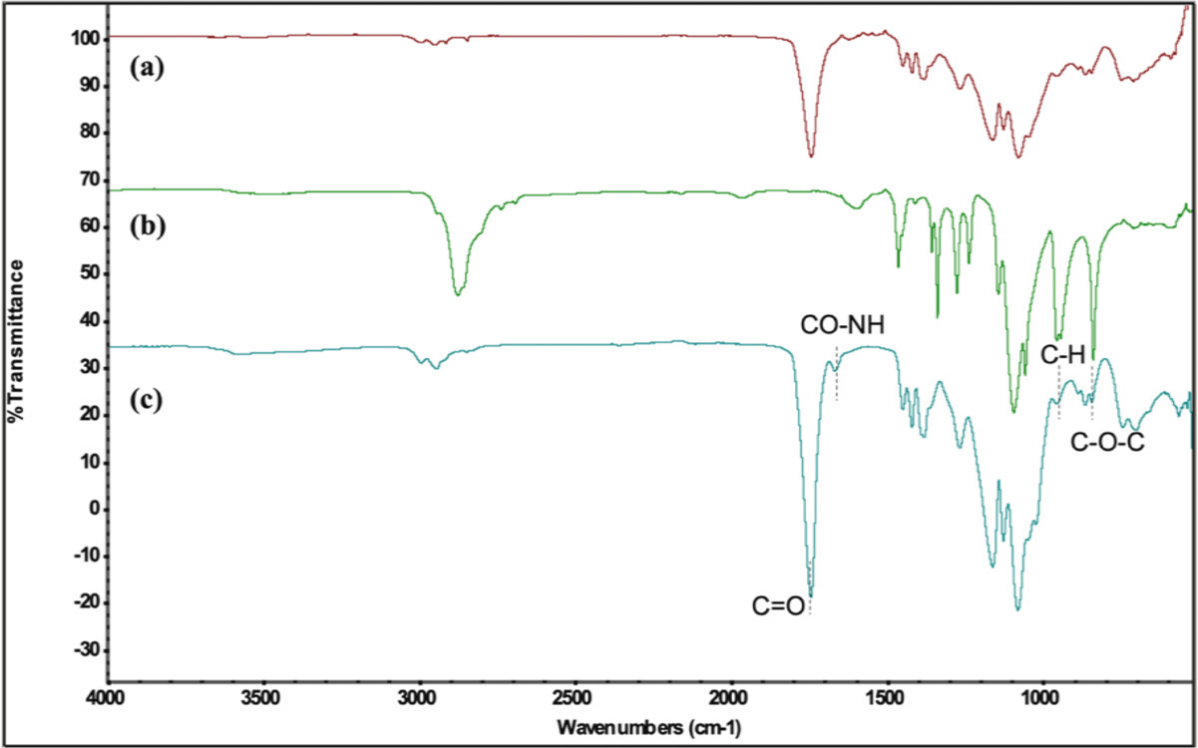
ATR-FTIR spectra of (a) PLGA, (b) PEG, and (c) PEGylated-PLGA polymers.

**Fig. 4:**
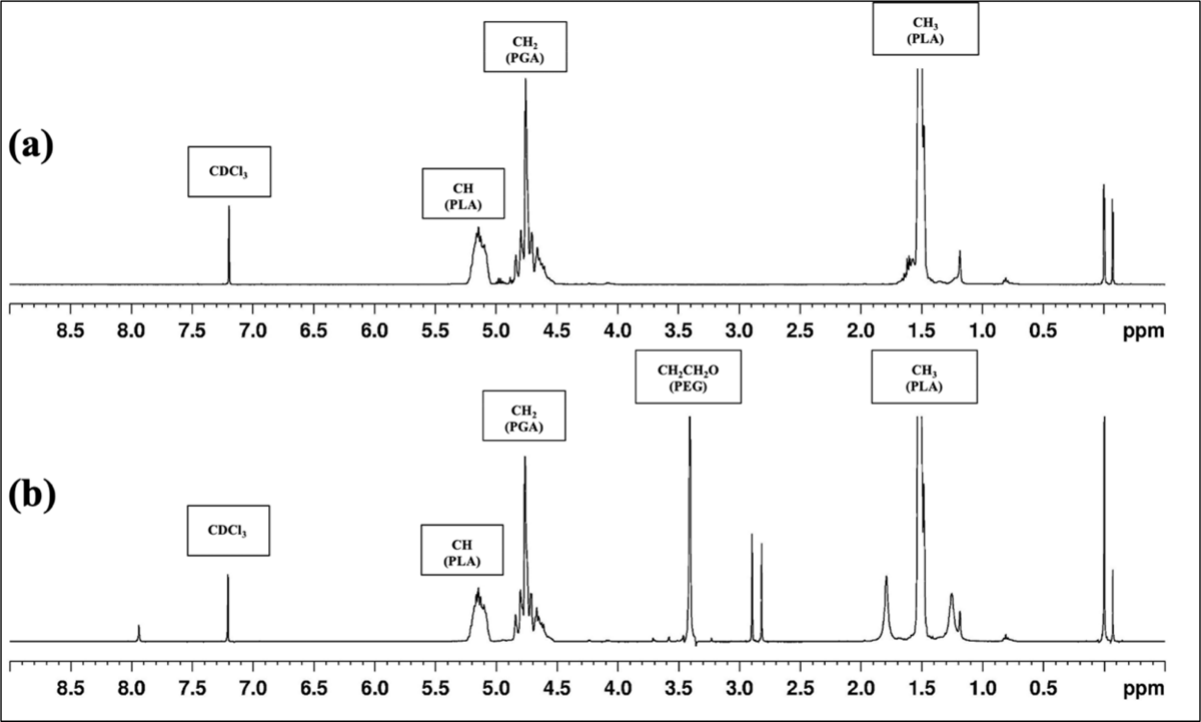
^1^H NMR spectra of synthetic (a) PLGA and (b) PEGylated-PLGA polymers.

The chemical synthesis of PEGylated-PLGA polymer was validated by ATR-FTIR (Fig. 3) and ^1^H NMR (Fig. 4) spectra. In Fig. 3c, the synthesized PEGylated-PLGA polymer showed a strong peak at 1746 cm^−1^ that represents the C=O stretching of PLGA. On the other hand, the existence of PEG is indicated by the appearance of tiny peaks at 950 cm^−1^ (bending vibration of C—H), and 841 cm^−1^ (stretching vibration of C—O—C). Furthermore, as indicated at 1673 cm^−1^, the amide's hydrogen-bonded carbonyl (CO—NH) was successfully created (Fig. 3c).

PEGylated-PLGA exhibited both PLGA and PEG spectral peaks on ^1^H NMR analysis. The generated PEGylated-PLGA contains peaks at 1.6 ppm (CH_3_), 4.8 ppm (CH_2_), and 5.2 ppm (CH), all of which reflect the PLGA structure (Fig. 4a). Additionally, the peak at 3.4 ppm (CH_2_CH_2_O) indicates the presence of PEG in the PEGylated-PLGA copolymer structure (Fig. 4b). The manufacturing efficiency (Eq. (1)) and PEGylation efficiency (Eq. (2)) of the PEGylated-PLGA copolymer, were calculated to be 80% and 50%, respectively. As proven by both ^1^H NMR and FTIR spectra, the PEGylated-PLGA polymer was synthesized successfully.

(1) $$\text{Manufacturing}\;\text{efficiency}\;\left(\%\right)=\frac{\text{Synthesized}\;\text{amount}\;\text{of}\;\text{PEGylated}-\text{PLGA}}{\text{Initial}\;\text{amount}\;\text{of}\;\text{PLGA}\;\text{and}\;\text{PEG}\;}$$

(2) $$\text{Pegylation}\;\text{efficiency}\;\left(\%\right)=\frac{{\left(\text{Peak}\;\text{area}\right)}_{\text{PEG}}/4}{\left[{\left(\text{Peak}\;\text{area}\right)}_{\text{PLA}}+{\left(\text{Peak}\;\text{area}\right)}_{\text{PGA}}+{\left(\text{Peak}\;\text{area}\right)}_{\text{PLA}}\right]/6}\;$$

**Fig. 5:**
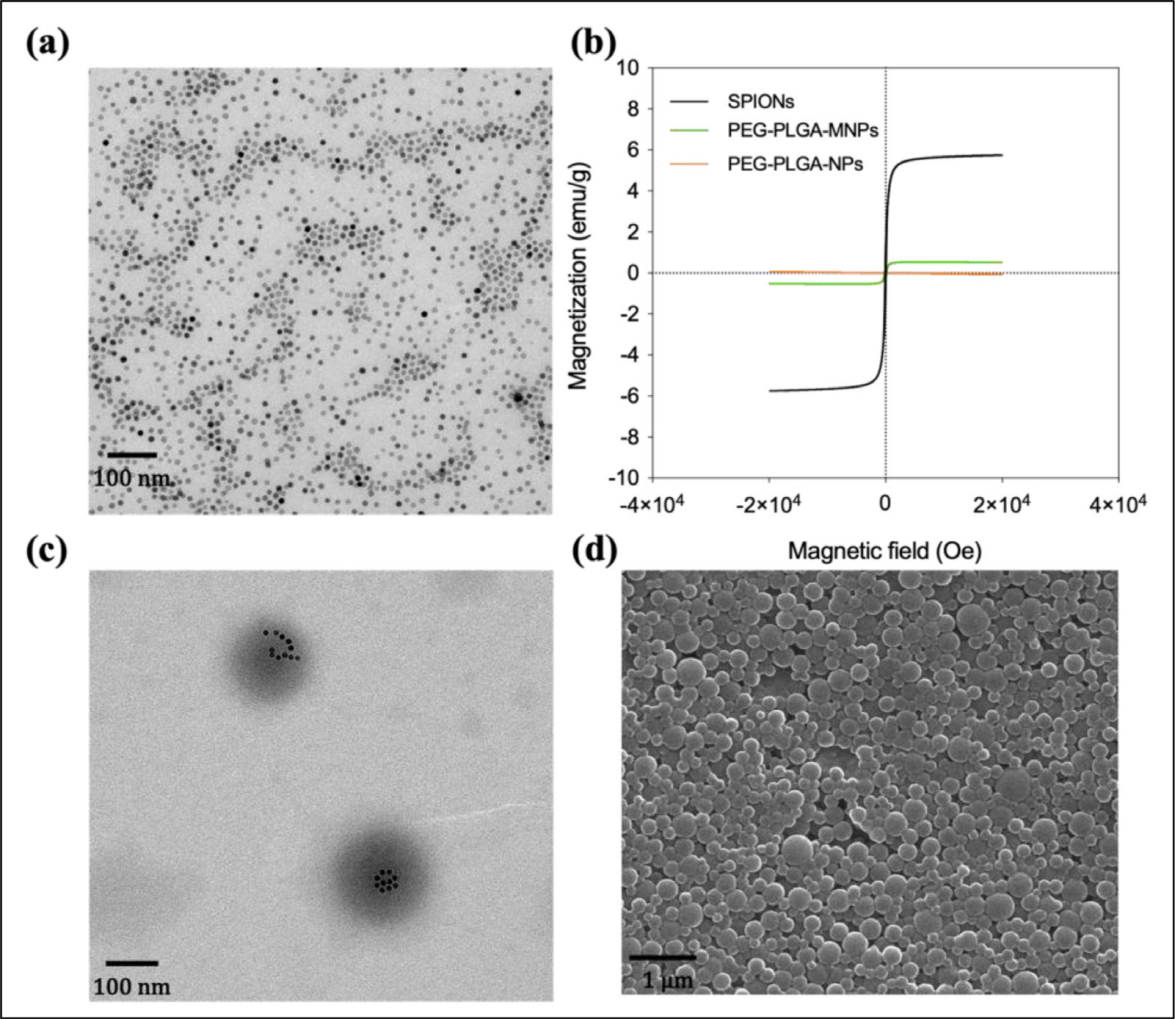
(a) TEM image of superparamagnetic iron oxide nanoparticles (SPIONs), (b) magnetic saturation curves for SPIONs, PEG-PLGA NPs and PEG-PLGA-MNPs, (c) TEM image of PEGylated-PLGA magnetic nanoparticles (PEG-PLGA MNPs), (d) SEM image of PEG-PLGA MNPs.

The size, morphology, and magnetic characteristics of superparamagnetic iron oxide nanoparticles (SPIONs) and magnetic PEGylated-PLGA nanoparticles (PEG-PLGA MNPs) were observed using transmission electron microscopy (TEM), scanning electron microscopy (SEM), and quantum design physical property measurement system (QD-PPMS) (Fig. 5). It was found that SPIONs, with an average size of around 5 nm, were highly monodisperse and had a narrow size distribution (Fig. 5a). A homogeneous distribution of SPIONs within the PEGylated-PLGA MNPs was also confirmed, indicating that the SPIONs were successfully encapsulated (Fig. 5c). In PEGylated-PLGA MNPs, SPIONs did not aggregate but instead acquired well-defined spherical shape (Fig. 5c). As illustrated in Fig. 5d, SPIONs-loaded PEGylated-PLGA MNPs are synthesized into a spherical shape with a mean diameter of 170 nm. Furthermore, all the samples' magnetization curves clearly display superparamagnetic behavior (SPIONs and PEG-PLGA-MNPs), with coercivity values that are near to zero (Fig. 5b). It was discovered that SPIONs had a saturation magnetization of 6.0 emu/g, and the potential causes of this low number are addressed in the magnetization hints section of SPIONs.

**Fig. 6:**
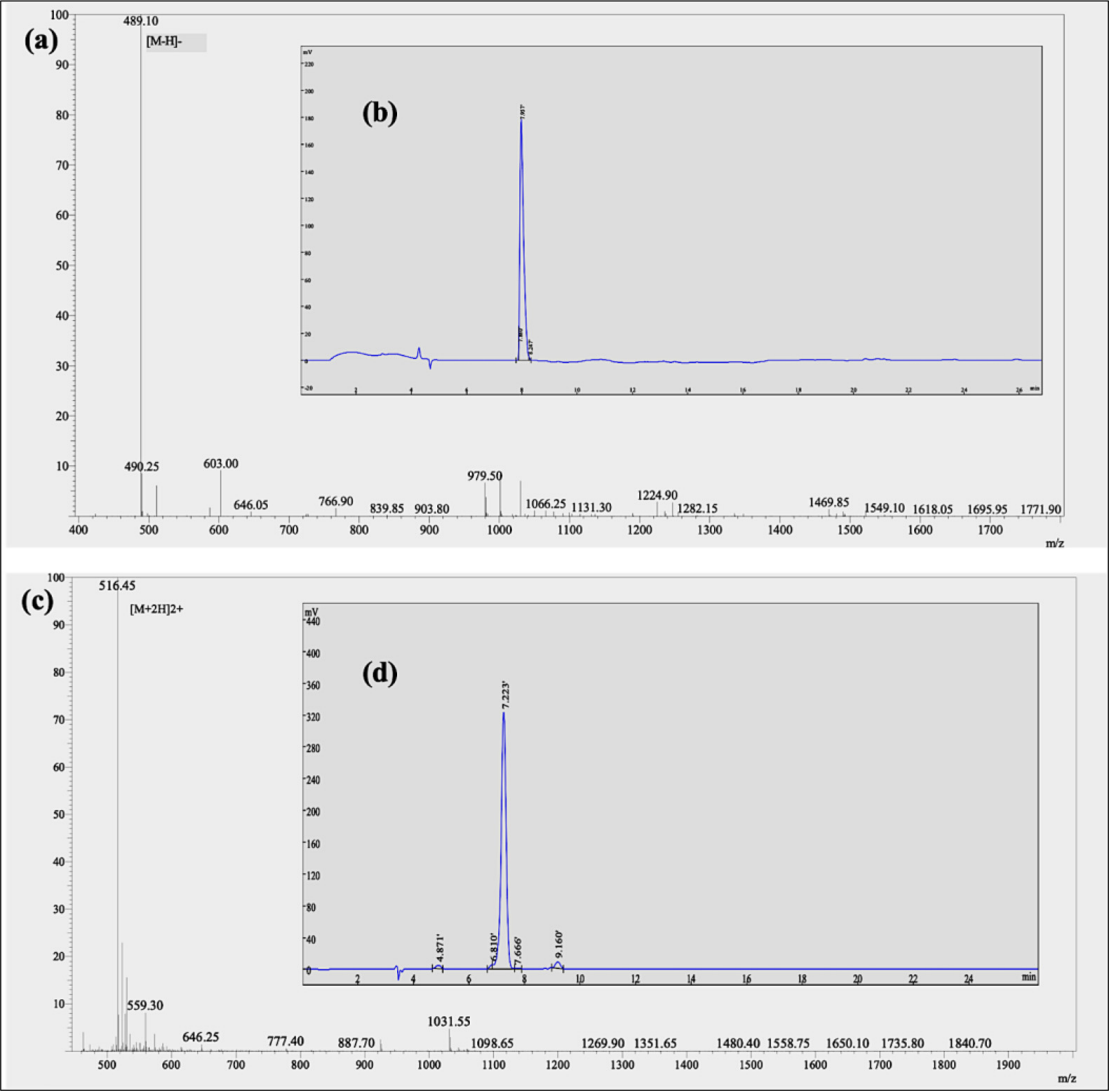
GRGDS peptide (a) mass spectrum and (b) HPLC chromatogram; TAMRA-GRGDS peptide (c) mass spectrum and (d) HPLC chromatogram.

After NPs were synthesized and characterized, GRGDS and TAMRA-GRGDS were chemically immobilized on PEG-PLGA-MNPs with or without curcumin by using EDC—NHS chemistry. The molecular weights of GRGDS and TAMRA-GRGDS peptides were determined by mass spectrometry to be 490.47 g/mol (Fig. 6a) and 1031.09 g/mol (Fig. 6c), respectively. These values are in accordance with the predicted molecular weights of the peptides. Additionally, HPLC analysis demonstrates that the GRGDS (99%, Fig. 6b) and TAMRA-GRGDS (95%, Fig. 6d) peptides are both highly pure, and they can be utilized directly for *in vitro* tests without the need for additional purification.

**Fig. 7:**
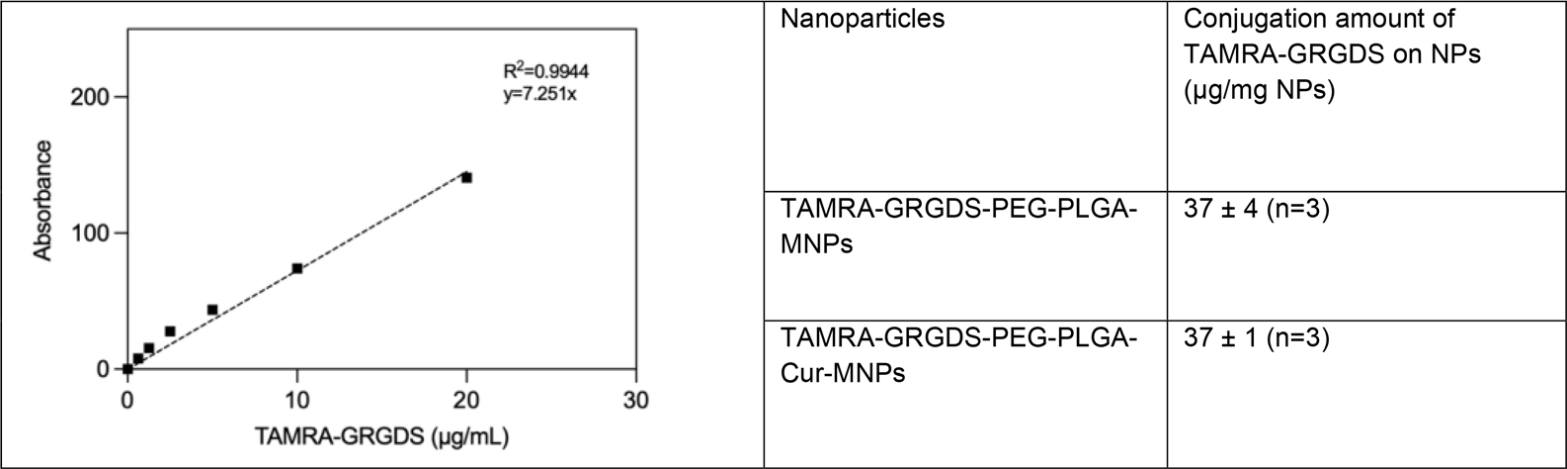
The fluorescence intensity of TAMRA-GRGDS (0–20 µg/mL) was measured at 544 nm excitation and 572 nm emission to generate the calibration curve. Thus, the amount of GRGDS-TAMRA bound to the nanoparticles (m-PNPs) was calculated.

We utilized a fluorescence spectrophotometer to indirectly detect the amount of GRGDS-TAMRA attached to the surface of NPs. For this reason, the fluorescence intensity of TAMRA-GRGDS was measured using excitation (544 nm) and emission (572 nm) wavelengths to generate the calibration curve (Fig. 7). Also, the conjugation quantity of GRGDS to the NPs was calculated to be 37 µg peptide/mg NPs. Therefore, it was proven that the GRGDS peptide had been effectively conjugated to NPs, ensuring the targeting capability for future tests.

**Fig. 8:**
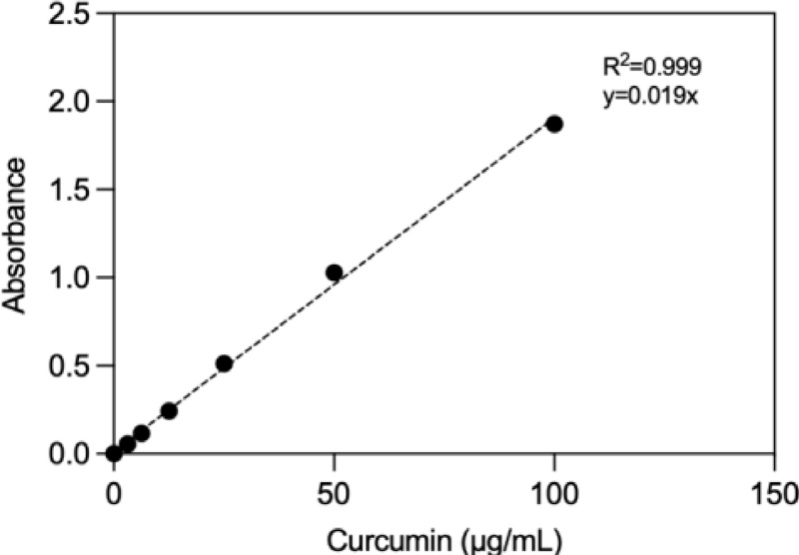
The calibration curve was obtained using standard solutions of curcumin dissolved in acetonitrile within 0–100 µg/mL range.

**Fig. 9:**
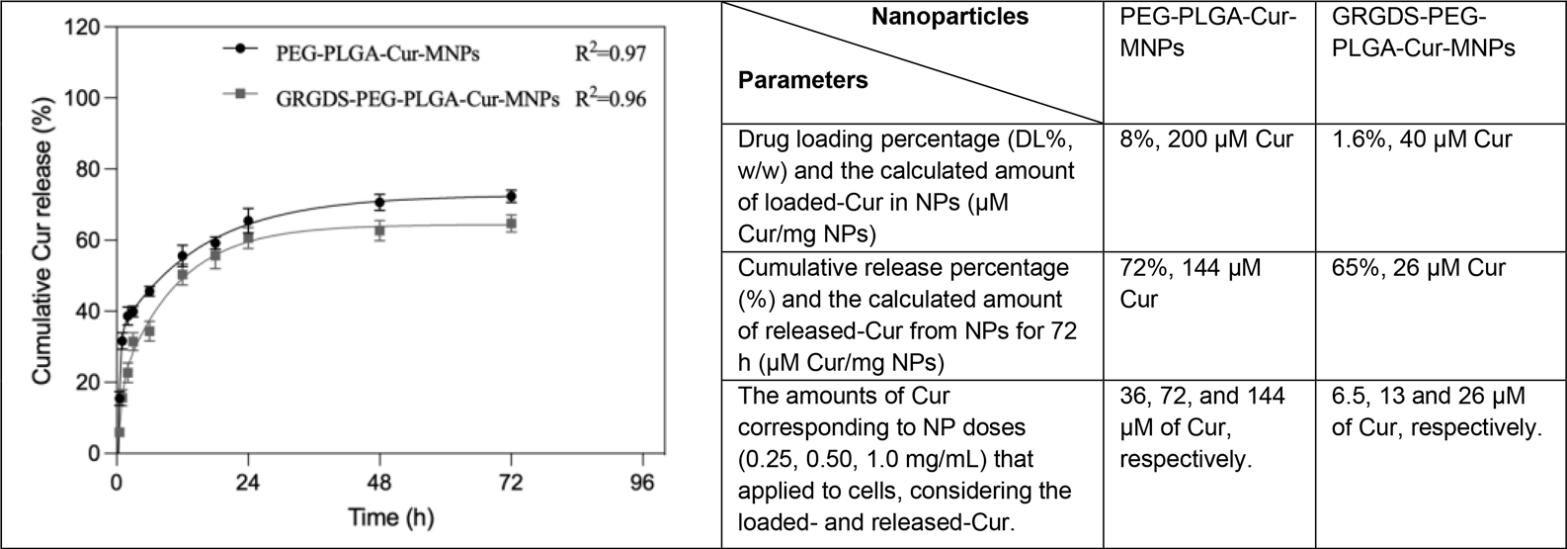
Curcumin release profiles from obtained nanoparticles were studied in PBS (pH=7.4) at 37 °C with a shaking incubator (100 rpm). All experiments were carried out in triplicate, and the mean ± SD was calculated. There are also provided estimated doses of curcumin that consider its parameters of loading (DL%, w/w) and 72 h release.

Encapsulation efficiency (EE%) and loading efficiency (DL%) of the curcumin were determined using the calibration curve (Fig. 8). The curcumin loading efficiency (w/w) of PEG-PLGA-Cur-MNPs (peptide-free) and GRGDS-PEG-PLGA-Cur-MNPs were calculated to be 8% and 1.6%, respectively. The difference in the amount of Cur loading between the PEG-PLGA MNPs with and without GRGDS is most likely the result of curcumin leaking from the NPs during their required washing and shaking steps for the peptide conjugation process. On the other hand, the release of Cur from PEG-PLGA-Cur-MNPs and GRGDS-PEG-PLGA-Cur-MNPs was 72% and 65%, respectively, during the period of the 72 h release (Fig. 9). In addition, the therapeutic effects of nanoparticles on human glioblastoma cells were analyzed after 72 h because of the continued curcumin release from nanoparticles for up to that period.

### A preliminary *in vitro* cytotoxicity test

**Fig. 10:**
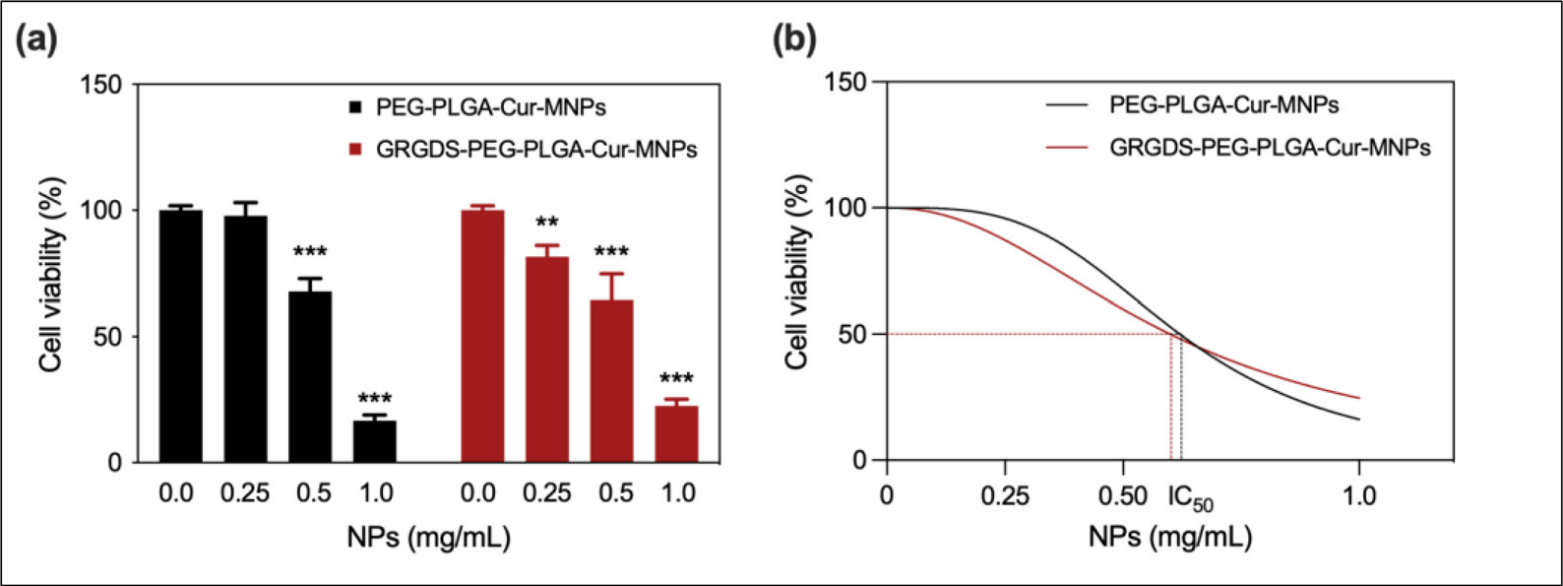
*In vitro* cytotoxicity of PEG-PLGA-Cur-MNPs and GRGDS-PEG-PLGA-Cur-MNPs on human glioblastoma cell lines (T98G) after 72 h of treatment. (a) Cytotoxic activity measurements of NPs were carried out by MTT, and data were represented as mean ± SD (*n* = 5), *p ≤ 0.05, **p ≤ 0.01, ***p ≤ 0.001. (b) The concentrations of both NPs that inhibited 50% of cell growth (IC_50_) were determined using a nonlinear regression curve fit by GraphPad Prism 9.5.0.

A preliminary cell viability test was conducted on T98G cells to compare the therapeutic efficacy of curcumin-loaded NPs with and without GRGDS (Fig. 10a). The cytotoxic effects of synthesized nanoparticles on human glioblastoma cells (T98G) were assessed by calculating their IC_50_ values (Fig. 10b), which were then compared based on the amount of loaded- and released- curcumin for 72 h. For instance, the IC_50_ values of NPs without and with GRGDS are 0.62 mg/mL (90 µM Cur) and 0.60 mg/mL (15 µM Cur), respectively. From this perspective, depending on the amount of loaded- and released- curcumin, the cytotoxicity of NPs with GRGDS was found to be about 6-fold higher after 72 h of treatment than that of NPs without GRGDS. The improved performance in the therapeutic efficacy of GRGDS-NPs was likely the result of the NPs' increased cellular uptake and targeting capabilities. Overall, we may conclude that GRGDS-conjugated NPs boosted the bioavailability of curcumin by increasing cellular intake through specific binding to cell receptors.

## Conclusion

This study includes the fabrication and characterization of PEGylated-PLGA nanoparticles functionalized with GRGDS peptides that can be used for cellular tracking and targeting purposes. These PEGylated-PLGA NPs were also evaluated as nanocarriers by loading with SPIONs and the natural compound curcumin. Following the fabrication of peptide-conjugated Cur-loaded nanocarriers, their effectiveness was assessed using a pilot cell viability assay with T98G cells. The most crucial result of our research is the promising use of GRGDS peptide as a targeting ligand which helps to decrease the active agent dose by 6-fold compared to peptide-free NPs. Considering this, the constructed nanocarrier system is particularly suitable for increasing the bioavailability of highly toxic drugs, and it can be adapted to other nanocarrier applications.

## CRediT authorship contribution statement

**Fatih Senturk:** Conceptualization, Methodology, Data curation, Formal analysis, Investigation, Writing – original draft, Visualization. **Soner Cakmak:** Conceptualization, Methodology, Writing – review & editing, Supervision, Project administration.

## Declaration of Competing Interest

The authors declare that they have no known competing financial interests or personal relationships that could have appeared to influence the work reported in this paper.

## Data Availability

Data will be made available on request.

## Appendix Supplementary materials

Image, application 1
